# Quorum sensing sets the stage for the establishment and vertical transmission of *Sodalis praecaptivus* in tsetse flies

**DOI:** 10.1371/journal.pgen.1008992

**Published:** 2020-08-14

**Authors:** Miguel Medina Munoz, Noah Spencer, Shinichiro Enomoto, Colin Dale, Rita V. M. Rio

**Affiliations:** 1 Department of Biology, Eberly College of Arts and Sciences, West Virginia University, Morgantown, WV, United States of America; 2 Department of Biology, University of Utah, Salt Lake City, UT, United States of America; Yale School of Public Health, UNITED STATES

## Abstract

Bacterial virulence factors facilitate host colonization and set the stage for the evolution of parasitic and mutualistic interactions. The *Sodalis-*allied clade of bacteria exhibit striking diversity in the range of both plant and animal feeding insects they inhabit, suggesting the appropriation of universal molecular mechanisms that facilitate establishment. Here, we report on the infection of the tsetse fly by free-living *Sodalis praecaptivus*, a close relative of many *Sodalis-*allied symbionts. Key genes involved in quorum sensing, including the homoserine lactone synthase (*ypeI*) and response regulators (*yenR* and *ypeR*) are integral for the benign colonization of *S*. *praecaptivus*. Mutants lacking *ypeI*, *yenR* and *ypeR* compromised tsetse survival as a consequence of their inability to repress virulence. Genes under quorum sensing, including homologs of the binary insecticidal toxin PirAB and a putative symbiosis-promoting factor CpmAJ, demonstrated negative and positive impacts, respectively, on tsetse survival. Taken together with results obtained from experiments involving weevils, this work shows that quorum sensing virulence suppression plays an integral role in facilitating the establishment of *Sodalis*-allied symbionts in diverse insect hosts. This knowledge contributes to the understanding of the early evolutionary steps involved in the formation of insect-bacterial symbiosis. Further, despite having no established history of interaction with tsetse, *S*. *praecaptivus* can infect reproductive tissues, enabling vertical transmission through adenotrophic viviparity within a single host generation. This creates an option for the use of *S*. *praecaptivus* in the biocontrol of insect disease vectors via paratransgenesis.

## Author summary

Symbiosis drives organismal novelty. Yet, we know little about the origin, establishment and persistence of symbiosis between animals and bacteria. *Sodalis*-allied symbionts have established independent infections in many insects. Here we show that quorum sensing facilitates the establishment of a novel *Sodalis praecaptivus* infection in tsetse flies, in a fashion very similar to that observed in weevils. Importantly, quorum sensing modulation of virulence allows *Sodalis* to establish in these hosts with minimal pathology and may explain the propensity for these symbionts to adopt associations with a wide range of hosts. Furthermore, *S*. *praecaptivus* infects reproductive tissues, enabling vertical transmission within a single host generation, potentially facilitating the use of *S*. *praecaptivus* in the control of insect disease vectors via paratransgenesis.

## Introduction

Bacteria occupy countless niches, including numerous adaptive (mutualistic) associations with plants and animals. Here, these microbes play numerous beneficial roles in host biology, impacting development [1,2], defense [3], and enhancing immunity and nutrition ([4], reviewed in [5]). Apart from a few examples provided by symbioses that rely on environment-mediated transmission, such as the *Vibrio*-squid and the *Rhizobium*-legume associations [6,7], molecular mechanisms that enable initial establishment and subsequent vertical transmission remain largely obscure [8]. Bacterial genera that are capable of infecting a wide range of arthropods and persisting by vertical transmission (such as *Wolbachia*, *Arsenophonus*, *Rickettsia* and *Sodalis* [9–12]) offer insight into these molecular features. Deciphering these key traits that facilitate host relations is useful for developing applications which require establishment and maintenance of genetically modified symbionts within a host, such as the use of probiotics to restore the composition of gut flora or the implementation of paratransgenesis to express novel genes in a host using genetically engineered symbionts [13,14].

Closely related *Sodalis-*allied symbionts have been identified within numerous insect orders. A spectrum of *Sodalis-*insect interactions ranging from enigmatic and facultative to mutualistic and obligate has arisen through multiple independent infection events [9,15–24]. Unlike other symbiont clades in which close non insect-associated members of the genus are not known, the genus *Sodalis* includes *S*. *praecaptivus*, which is viewed and has been experimentally adopted as a closely-related environmental antecedent to the *Sodalis-*allied symbionts found in many insect taxa [25]. *S*. *praecaptivus* was cultured fortuitously from a human hand infection following impalement with a dead tree branch [25]. This bacterium was identified using 16S rRNA analysis that revealed high sequence identity (99%) to the recently derived endosymbionts of various weevil and stink bug species [25]. The identification of an environmental reservoir for *Sodalis-*allied bacteria further supports a source-sink model [26] for the transfer of a free-living bacterium (i.e. source) into diverse insect hosts (i.e. sinks) in which mutualistic associations can then evolve. Importantly, the capacity to maintain *S*. *praecaptivus* in culture coupled with its amenability towards genetic manipulation provides an ideal model to further our understanding of key factors enabling the commencement and progression of symbioses in a wide range of insects.

The *S*. *praecaptivus* genome [25] is considerably larger than other *Sodalis*-allied symbionts and contains substantially more intact homologs of virulence and toxin genes that are typically associated with animal and plant pathogens. The reduced genomes of the *Sodalis-*allied endosymbionts are subsets of *S*. praecaptivus, derived following transition to restricted insect-associated lifestyles. Thus, the larger gene inventory found in *S*. *praecaptivus* is thought to allow greater environmental and host plasticity, some of which may facilitate host-specific exchanges instrumental towards the establishment of insect symbioses. Interestingly, *S*. *praecaptivus* is able to colonize within grain weevils through the use of quorum sensing that limits the expression of virulent insecticidal genes to only within the incipient stages of infection enabling population growth and persistence by gaining access to host tissues and cells [27].

Quorum sensing is a form of intercellular communication that enables bacteria to determine their local population density, and coordinate collective group behavior such as swarming, motility and light production, through the simultaneous regulation of gene expression across the population [28,29]. Quorum sensing is directed by the synthesis and subsequent diffusion of small molecules known as autoinducers (e.g. N-acyl homoserine lactone signaling molecules for some Gram-negative bacteria) that can diffuse in and out of the bacterial cell and modulate transcription of target genes via interaction with specific protein response regulators [30]. The disruption of quorum sensing within *S*. *praecaptivus* results in a rapid and potent host killing phenotype upon microinjection into weevils [27]. This occurs because quorum sensing normally represses the expression of virulence factors associated with insect killing, including the PirAB insecticidal toxins, chitinases and collagenase-like proteins [27]. Under natural circumstances, these virulence factors are expressed only at low bacterial population density (i.e. during the initial stage of infection), presumably to allow bacteria to gain access into host tissues and cells [27].

While the results obtained from studies involving weevils are intriguing, they currently represent an isolated case and the significance of the quorum sensing control of virulence towards the colonization of *S*. *praecaptivus* in a broader range of insects has not been explored. Here, we examine the ability of *S*. *praecaptivus* to establish in a member of a distinct order of insects, the Dipteran tsetse fly *Glossina morsitans*, which also harbors an autochthonous *Sodalis-*allied symbiont (*S*. *glossinidius*). The colonization, persistence and localization of *S*. *praecaptivus* within tsetse flies were evaluated relative to bacterial population density and host life history traits, specifically lifespan and fecundity. We find that key genes in quorum sensing, including those encoding a homoserine lactone synthase (*ypeI*) and response regulators (*yenR* and *ypeR*) also play an integral role in virulence restraint in tsetse, with *S*. *praecaptivus* mutants (lacking these genes) producing significantly greater tsetse mortality following their introduction. These results are consistent with the notion that quorum sensing functions to control the virulence of *Sodalis* in a broad range of insects. Our work also shows that, despite having no established history of interaction with tsetse, *S*. *praecaptivus* infects tsetse reproductive tissue, enabling vertical transmission within a single host generation. The amenability of *S*. *praecaptivus* towards genetic engineering [27], its ease of establishment within tsetse coupled with its ability to be vertically transmitted (albeit currently at low levels), provides a new option for the development and implementation of paratransgenic tools for disease control, perhaps including tsetse transmitted trypanosomiasis. In addition to enhancing our understanding of molecular features involved in the initiation of symbiosis, *S*. *praecaptivus* also provides a model for future studies of the mechanistic basis of vertical transmission, which is poorly understood in insects.

## Results

### *S*. *praecaptivus* establishes a persistent and benign infection within tsetse

Successful establishment of a microbe in a host is dependent on interactions with immunological responses, nutritional resources, and resident microbiota [23,26,31]. It was previously shown that *S*. *praecaptivus*, a free-living relative of the *Sodalis*-host associated symbionts found within many different insects, was capable of sustaining infections within *Si*. *zeamais* grain weevils cleared of their endogenous *Ca*. S. pierantonius symbionts [27]. Here, we subjected tsetse flies to a similar infection regimen to determine if they could also be infected with *S*. *praecaptivus*. In this case, *S*. *praecaptivus* was introduced into flies either *per os* in heat-inactivated (HI) blood or by intrathoracic microinjection at concentrations similar to those previously used for the inoculation of foreign bacteria into tsetse [27,32] (Table 1).

**Table 1:** *S*. *praecaptivus* infection of tsetse. The percentage of tsetse infected with WT *S*. *praecaptivus* following either introduction through *per os* supplementation of heat inactivated blood meal or thoracic microinjection. *G*. *morsitans*^STZ^ = *S*. *glossinidius-*free *G*. *morsitans*. Fly infection status was determined 7 days following WT (CD14) *S*. *praecaptivus* introduction.

Mode of introduction	CFU introduced per fly/ tsetse line	% of flies infected (confirmed infections/flies challenged)
*per os*	1x10^3^ /*G*. *morsitans*	79% (107/135)
	1x10^5^ /*G*. *morsitans*	87% (39/45)
	1x10^3^ /*G*. *morsitans*^STZ^	75% (9/12)
	1x10^5^ / *G*. *morsitans*^STZ^	100% (12/12)
microinjection	1x 10^5^ /*G*. *morsitans*	100% (63/63)
	1x10^5^ / *G*. *morsitans*^STZ^	89% (8/9)

**Fig 1:**
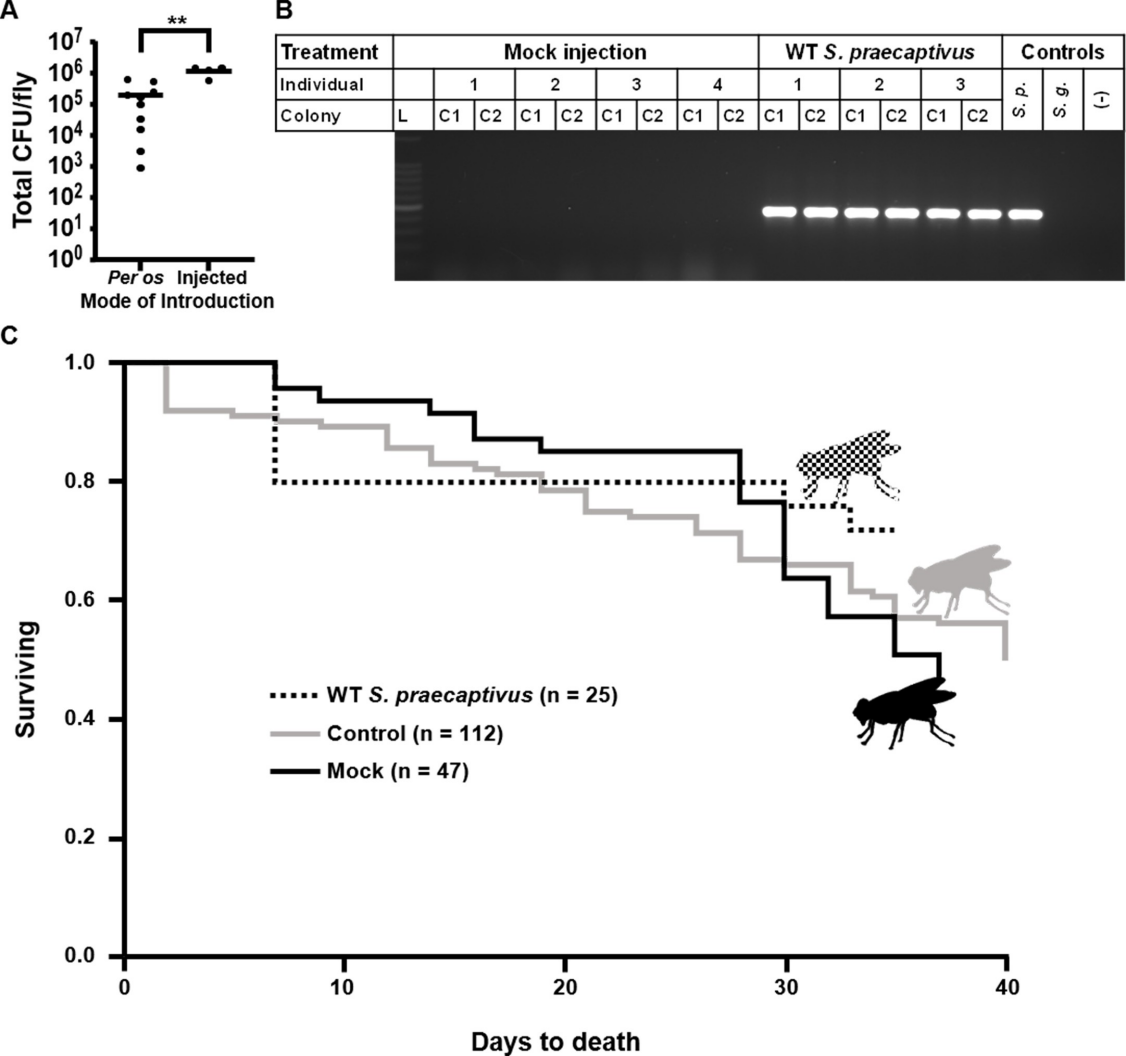
*S*. *praecaptivus* can establish long lasting tsetse infections. A. Total WT *S*. *praecaptivus* density per fly is greater when individuals are challenged by microinjection versus *per os*. Both groups were administered 1 x 10^5^ bacteria. The density of WT *S*. *praecaptivus* flies following 7 d post introduction was assessed via CFU counts after surface sterilization, homogenization, serial dilution and plating. Horizontal lines represent mean bacterial count per treatment, while dots indicate the bacterial density within individual flies. The Y axis (Total CFU/fly) is plotted in logarithmic scale. Mean bacterial density between introduction modes was compared through a Mann-Whitney U test (**, *p* = 0.004). B. PCR for the *S*. *praecaptivus* specific *tam* gene (Genbank: AHF76984.1, 442 bp amplicon) confirmed that colonies from plating of WT *S*. *praecaptivus-*injected fly homogenates 50 days post microinjection were *S*. *praecaptivus*, indicating persistent infections. Sampled colonies from mock-injected fly homogenates showed no evidence of *S*. *praecaptivus* infection. Individual = distinct flies; Colony = two bacterial colonies per fly were randomly selected for PCR verification; L = DNA molecular marker; *S*.*p*. = *S*. *praecaptivus* DNA; *S*.*g*. = *S*. *glossinidius* DNA; (-) = negative control corresponding to a no DNA template reaction. This assay was carried out for > 10 individuals per group, and one representative analysis is shown here. C. The established WT *S*. *praecaptivus* infection does not impact tsetse survival. Kaplan-Meier curves comparing survival of tsetse injected with *S*. *praecaptivus* to those of noninjected and mock injected control lines. During the observation period there were no significant differences between the survival of control flies and those microinjected with WT *S*. *praecaptivus* (*S*. *praecaptivus* vs. mock injected; Log-rank test, *p* = 0.1526, *S*. *praecaptivus* vs. control [non-injected flies]; Log-rank test, *p* = 0.2250). n = flies per treatment group. WT *S*. *praecaptivus* used was CD14 (S1 Table).

Tsetse flies were examined 7 d following bacterial challenge for the presence of infection by plating surface-sterilized homogenized flies and PCR amplifying the DNA of resulting bacterial colonies with *S*. *praecaptivus*-specific trans-aconitate 2-methyltransferase (*tam*) gene primers. Tsetse flies were found to be capable of sustaining *S*. *praecaptivus* infections throughout our initial (7d) observation window using both *per os* (~80% of tsetse individuals receiving a 1 x 10^3^ CFU introduction of WT *S*. *praecaptivus* harbored infections while a slightly higher infection rate of 87% was achieved when challenging tsetse individuals with a 1 x 10^5^ CFU introduction of WT *S*. *praecaptivus*, Table 1) and intrathoracic microinjection (100% of challenged tsetse individuals were infected at 7 d with introduction of 1 x 10^5^ CFU of WT *S*. *praecaptivus*, Table 1). Microinjection into tsetse hemolymph proved slightly more efficacious towards *S*. *praecaptivus* establishment supported by the higher prevalence of infection among individuals in comparison to oral introduction (100% versus 87% of individuals at a 1 x 10^5^ CFU delivery; respectively, Table 1). The microinjection of *S*. *praecaptivus* also resulted in significantly greater CFU per fly in comparison to *per os* at 7 d post introduction (Fig 1A, Mann-Whitney U test, *p* = 0.004). Furthermore, infections were found to persist throughout the tsetse lifespan as *S*. *praecaptivus* presence was observed at 50 d post microinjection (Fig 1B). The *S*. *praecaptivus* infections also appear to be benign with respect to host lifespan, as no adverse effects towards tsetse survival were observed when compared to noninjected (Log-rank test, *p* = 0.2250) and mock-injected controls (Log-rank test, *p* = 0.1526) (Fig 1C).

### Quorum sensing is essential for *S*. *praecaptivus* persistence within tsetse

In *S*. *praecaptivus*, the homoserine lactone (3-oxo-hexanoyl homoserine lactone; OHHL) is synthesized by a homolog of the canonical LuxI synthase, designated YpeI, where it is sensed by two response regulator homologs, YpeR and YenR [27]. When OHHL density surpasses a threshold, *ypeR* and *yenR* modulate gene expression profiles at the population level, downregulating the expression of virulent toxins and preventing host killing [27]. Based on a study involving weevils, it was proposed that the quorum sensing system in *S*. *praecaptivus* might play a key role in allowing the bacterium to establish asymptomatic infections in a broad range of insect hosts [27]. This would be anticipated to be adaptive under circumstances in which these bacteria are transmitted (between plant and/or animal hosts) by insect vectors. In addition, it was proposed that it might underlie the propensity for these bacteria to adopt, permanent, mutualistic associations with a wide range of insect hosts. In order to evaluate the latter hypothesis we sought to determine if quorum sensing plays an important role in modulating the virulence of *S*. *praecaptivus* towards tsetse flies, which are representatives of an order of insects (Diptera) that is distantly related to weevils (Coleoptera). To determine the importance of quorum sensing for the successful establishment and persistence of *S*. *praecaptivus* within tsetse, several mutant strains with disruptions in key quorum sensing genes including *ypeI*, *ypeR*, and *yenR* were introduced into tsetse flies and their survival was monitored over time. A mutant lacking *ypeI*, along with the insecticidal binary toxin genes *pirA* and *pirB* [33], and a regulatory gene, *regC*, that seems to enhance *pirAB* expression (based on transcriptomic analyses) [27], was also introduced into tsetse to further assess the relationship between quorum sensing, the production of virulence factors, and tsetse fly survival.

**Fig 2:**
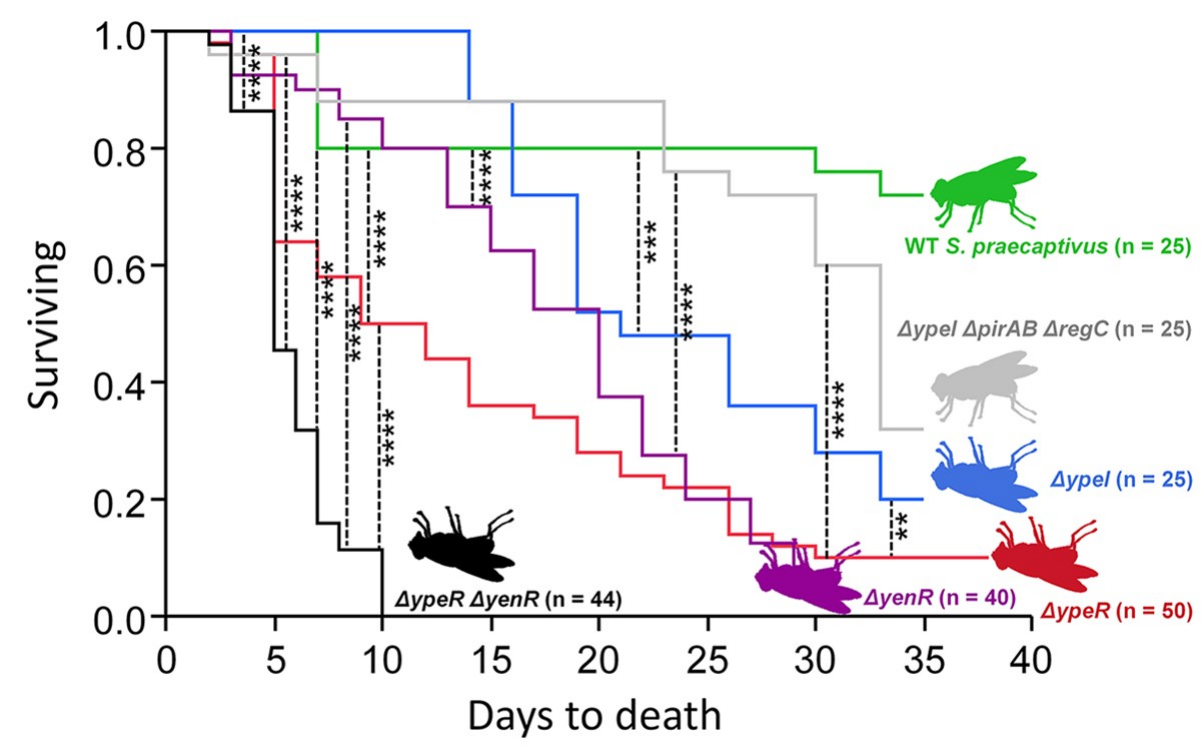
The disruption of *S*. *praecaptivus* quorum sensing (QS) genes decreases tsetse survival upon infection. Kaplan-Meier curves comparing survival of tsetse lines injected with *S*. *praecaptivus* mutants in QS genes. Survival curve of a tsetse line injected with wildtype *S*. *praecaptivus* is included for reference; log-rank test *p*-values are indicated for the corresponding pairwise comparisons between curves. Only statistically significant differences in tsetse survival upon microinjection of *S*. *praecaptivus* lines are indicated with asterisks signifying; ** *p* ≤ 0.01, *** *p* ≤ 0.001, and **** *p* ≤ 0.0001. WT alone: WT *S*. *praecaptivus*; *ΔypeR*: mutant of the LuxR-like response regulator gene *ypeR*; *ΔypeI*: mutant with an inactivation of the OHHL synthase gene *ypeI*; *ΔyenR*: mutant of the response regulator gene *yenR*; *ΔypeR ΔyenR*: mutant in both the response regulator genes *ypeR* and *yenR*; *ΔypeI ΔpirAB ΔregC*: quadruple disruption of OHHL synthase gene *ypeI*, homologs of the *pirA* and *pirB* genes coding for a binary insecticidal toxin, and the homolog of a transcriptional regulator of *pirAB*, *regC*. n = number of flies injected per treatment. WT *S*. *praecaptivus* used was CD14 (S1 Table).

Microinjection of a mutant lacking the gene encoding the homoserine lactone synthase, *ΔypeI*, caused a significant increase in fly mortality in comparison to flies microinjected with WT *S*. *praecaptivus* (Log-rank test; *p =* 0.0009, Fig 2). Further, flies that were challenged with mutants lacking response regulators, *ΔypeR* or *ΔyenR*, exhibited even greater mortality in a shorter span of time in comparison to flies injected with WT *S*. *praecaptivus* (Log-rank test; *p* <0.0001). However, the most deleterious effect on fly survival arose from a microinjection of a *S*. *praecaptivus* double mutant in the response regulators *ΔyenR* and *ΔypeR* (Log-rank test; p <0.0001, Fig 2) with exacerbated mortality exemplified by no tsetse fly surviving past 10 d post introduction. In contrast, flies injected with the *ΔypeIΔpirABΔregC* quadruple mutant showed enhanced survival in comparison to the isogenic *ΔypeI* mutant alone (Log-rank test; *p* = 0.05). This “rescue” effect, occurring when virulence genes that are regulated by quorum sensing are deleted, closely resembles the results obtained in the previous study on weevils [27], suggesting that the quorum sensing that *S*. *praecaptivus* uses to control insect infection and the toxins that engender killing may have wide applicability in insect hosts and, intriguingly, may also provide novel broad spectrum candidates for pest control. In addition, “rescue” from killing was also observed when a *ΔypeI* mutant was co-injected with an equivalent amount of WT *S*. *praecaptivus* (S1 Fig; Log-rank test; *p* < 0.0001). This can be explained as a consequence of the WT strain producing sufficient OHHL to induce quorum in the *ΔypeI* mutant strain, preventing it from producing the virulence factors that normally lead to host killing. To further disentangle the mechanisms operating to kill tsetse when quorum sensing genes are perturbed, *S*. *praecaptivus* abundance was determined in flies inoculated with either WT, *ΔypeI* or the *ΔypeIΔpirABΔyregC* quadruple mutant (S2 Fig). Flies injected with the *ΔypeI* mutant had a significantly greater abundance of *S*. *praecaptivus* relative to those injected with the WT strain (ANOVA, Tukey’s multiple comparisons test; *p* = 0.002), consistent with increased pathogenesis. In support, flies injected with *ΔypeIΔpirABΔyregC* mutants had *S*. *praecaptivus* densities that were in between those observed for WT and *ΔypeI* mutant strains. However, it is also possible that inappropriate regulation of QS virulence genes is causing pathogenesis, independent of (or in conjunction with) the increase in growth.

### The impact of *S*. *glossinidius* towards *S*. *praecaptivus* prevalence and density within tsetse

We were also interested in determining the impact of autochthonous *S*. *glossinidius* symbionts towards *S*. *praecaptivus* establishment within tsetse flies, particularly because *S*. *glossinidius* is known to have retained the capability to synthesize OHHL [34]. The specific removal of *S*. *glossinidius* (and not the obligate mutualist *Wigglesworthia glossinidia*) was achieved by maintaining tsetse flies on blood supplemented with streptozotocin (2-deoxy-2-(3-methyl-3nitrosoureido)-D-glucopyranoside) [35]. Streptozotocin is a bactericidal analogue of *N-*acetyl-D-glucosamine, the principle carbon source used for *S*. *glossinidius* growth within tsetse [36]. Progeny deposited by streptozotocin flies were verified to be *S*. *glossinidius*-free and used for the microinjection of *S*. *praecaptivus* (S3 Fig). The absence of endogenous *S*. *glossinidius* populations did not affect the infection prevalence of tsetse with *S*. *praecaptivus* when introduced either *per os* or via microinjection. Similar infection rates were observed when comparing the prevalence of *S*. *praecaptivus* colonization following microinjection into *G*. *morsitans*^WT^ (harboring *S*. *glossinidius*) or into individuals lacking *S*. *glossinidius* (*G*. *morsitans*^STZ^) (100% versus 89%, respectively; Table 1). The *per os* introduction of *S*. *praecaptivus* into these two populations of flies also yielded similar establishment rates (80% versus 75% with a 10^3^ CFU/fly introduction and 87% versus 100% with a 10^5^ CFU/fly introduction, respectively; Table 1). These results indicate that endogenous *S*. *glossinidius* neither facilitates nor impairs *S*. *praecaptivus* colonization of tsetse. Future studies should further explore this question of facilitation or inhibition by the endogenous *S*. *glossinidius* through testing a range of infectious doses, particularly at lower abundances (i.e. ID_50_) which may enable better identification of subtle differences in *S*. *praecaptivus* colonization.

**Fig 3:**
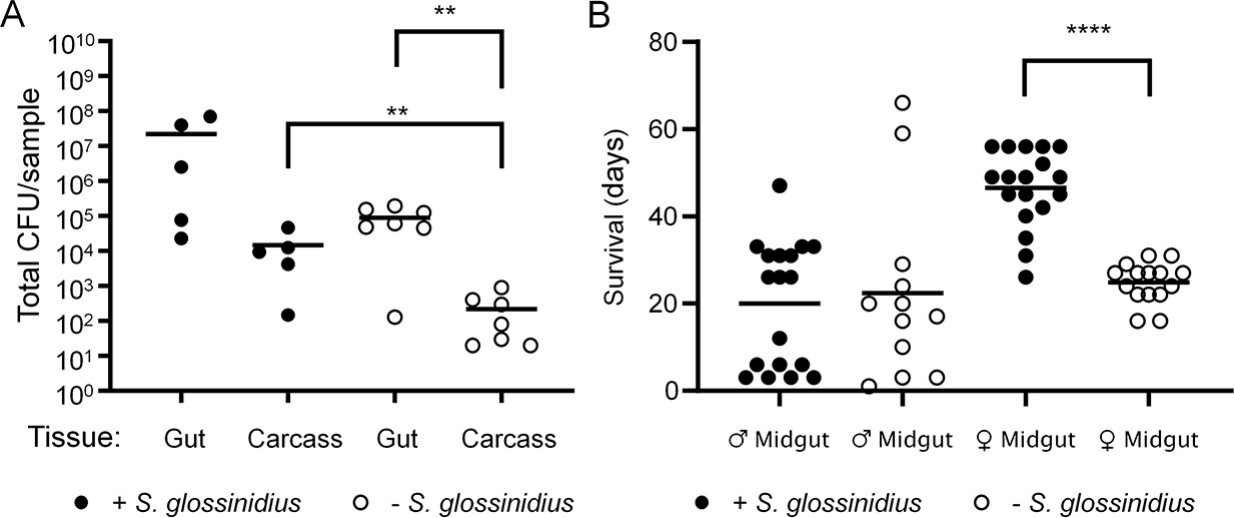
The effects of *S*. *glossinidius* on *S*. *praecaptivus* density and distribution within the tsetse fly and towards the complementation of the *S*. *praecaptivus ypeI* mutant. A. Comparison of *S*. *praecaptivus* abundance and distribution in flies seven days post introduction, with or without autochthonous *S*. *glossinidius*. All groups were injected with WT *S*. *praecaptivus*. The density of *S*. *praecaptivus* is significantly higher in the gut, relative to the carcass, when *S*. *glossinidius* is lacking within tsetse. The density of *S*. *praecaptivus* was significantly higher in tsetse carcass that harbored native *S*. *glossinidius* in comparison to flies that lacked this endosymbiont. Horizontal lines represent mean CFU per treatment, while dots indicate the bacterial CFU within individual flies. B. Endogenous *S*. *glossinidius* complements *S*. *praecaptivus ΔypeI* within females. Fly groups were injected with *S*. *praecaptivus ΔypeI* mutant and lifespan assessed per treatment group. Females showed a significant increase in lifespan when *S*. *glossinidius* is present upon the introduction of *S*. *praecaptivus ΔypeI* mutant. Horizontal lines represent mean survival (days) per treatment, while dots indicate the survival of individual flies, ** *p* ≤ 0.01 and **** *p* ≤ 0.0001. WT *S*. *praecaptivus* used was CD623 (S1 Table).

We then reasoned that even if *S*. *glossinidius* did not impact the establishment of infection, the density and distribution of *S*. *praecaptivus* might vary in the presence/absence of *S*. *glossinidius*. An increase in *S*. *praecaptivus* density within flies that harbored their endogenous *S*. *glossinidius* would suggest synergism between these two bacteria, while a decrease may point to competitive inhibition. To determine this, we compared the densities of *S*. *praecaptivus* in the midgut and carcass of the flies (defined as the whole fly minus the gastrointestinal tract from the anterior midgut to the malpighian tubules), comparing wild type (+Sg) tsetse with streptozotocin-treated individuals lacking *S*. *glossinidius* (-Sg) following microinjection. Only -Sg flies harbored significantly higher *S*. *praecaptivus* densities in their guts relative to carcasses (Fig 3A, Mann-Whitney U test, *p =* 0.0022). Surprisingly, there was a significantly greater density of *S*. *praecaptivus* detected in the carcasses of +Sg flies relative to -Sg flies (Fig 3A, Mann-Whitney U test, *p =* 0.01). Tsetse midguts harboring *S*. *glossinidius* also contained higher *S*. *praecaeptivus* densities relative to–Sg midguts, but lacked statistical significance (Fig 3A, Mann-Whitney U test, *p =* 0.20). This is difficult to explain on the basis that these two bacteria might be expected to compete for resources. The observed synergistic interaction certainly merits further exploration.

Since *S*. *glossinidius* and *S*. *praecaptivus* use the same signaling molecule (OHHL) to control their quorum sensing systems [27,34], we examined the ability of endogenous *S*. *glossinidius* to complement the *S*. *praecaptivus ΔypeI* mutant. We hypothesized that if the *S*. *praecaptivus* homoserine lactone synthesis (*ΔypeI*) mutant could be complemented by endogenous *S*. *glossinidius* OHHL then flies that harbor *S*. *glossinidius* might be rescued from killing mediated by the *S*. *praecaptivus ΔypeI* mutant, in the same way that killing is suppressed when the *ΔypeI* mutant is co-injected with WT (S1 Fig). Notably, following infection with the *S*. *praecaptivus ΔypeI* strain, female flies harboring *S*. *glossinidius* had a significantly longer lifespan then individuals lacking *S*. *glossinidius*, (Fig 3B, 46.5 ± 2.1 d versus 24.8 ± 1.2 d, respectively, Mann-Whitney U test, *p* < 0.0001). However, this effect was not observed in male flies, with members of both the -Sg and +Sg groups showing a similar mean longevity (Fig 3B, 19.9 ± 3.4 d versus 22.3 ± 6.0 d for +Sg males and -Sg males; respectively, Mann-Whitney U test, *p* = 0.6825). This difference may be explained by the fact that female tsetse harbor significantly higher densities of *S*. *glossinidius* [37], perhaps yielding sufficient (*S*. *glossinidius*-derived) OHHL to suppress killing by the *ΔypeI* mutant strain. Further, it is possible that the difference in survival observed between the sexes may be a function of the different lifespans of males and females [38], where the significantly shorter life span of males limits our ability to detect a rescue effect.

### *S*. *praecaptivus* infections impact tsetse fecundity

**Fig 4:**
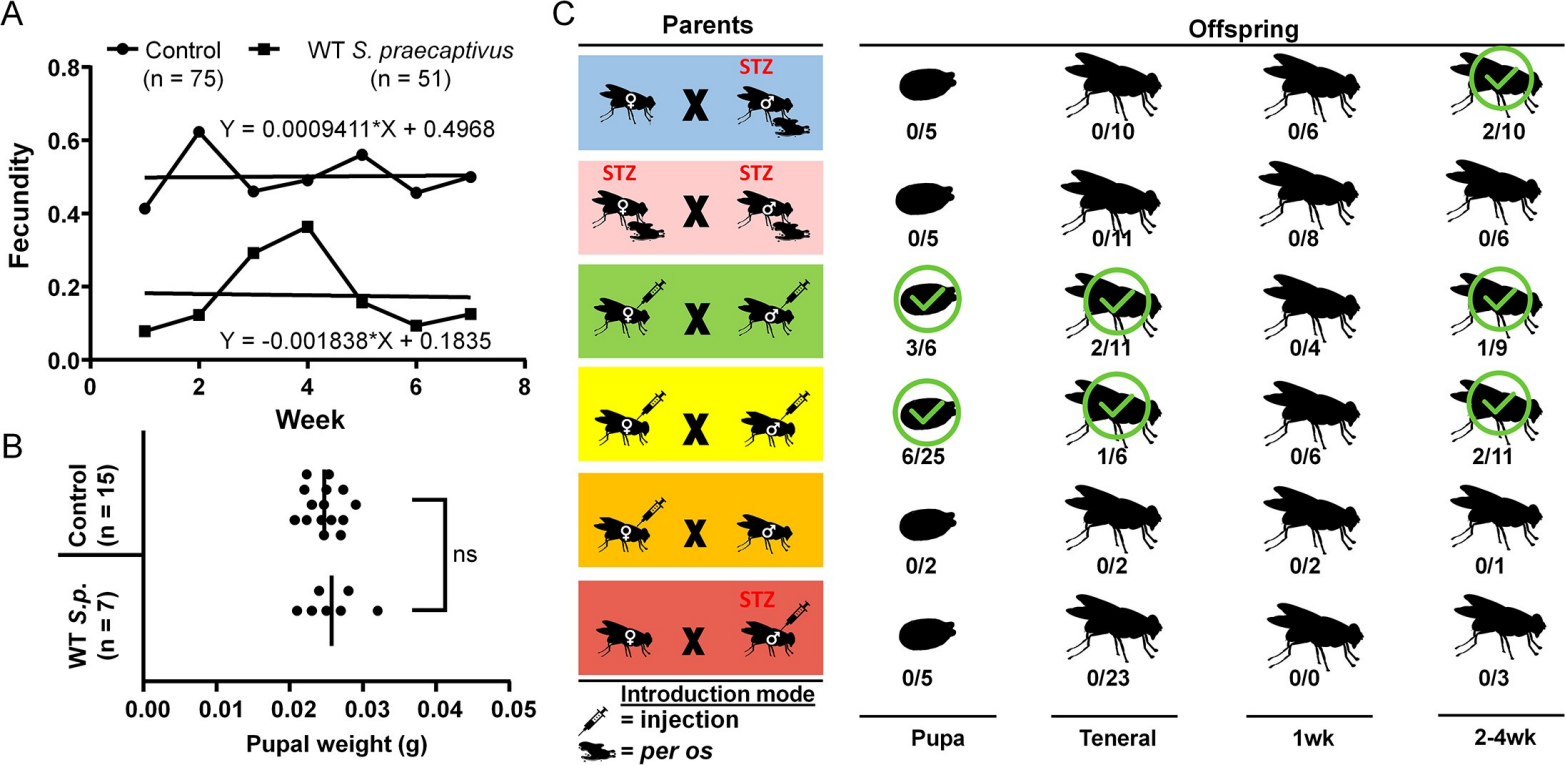
*S*. *praecaptivus* is vertically transmitted in tsetse but infection incurs a reproductive output cost. A. WT *S*. *praecaptivus* infected flies are fertile. Multiple linear regression analysis shows that fecundity of both control (n = 75) and infected (n = 51) flies commence at equivalent times remaining consistent through time, however; the reproductive output of flies infected with *S*. *praecaptivus* is lower. B. There was no significant difference in the weight of pupae deposited by *S*. *praecaptivus* infected flies upon comparison to pupae deposited by age matched control flies. Vertical bars indicate the mean pupal weight (grams) within a treatment group, each dot represents an individual pupa. C. Infection status at different life stages of progeny arising from different crosses of tsetse individuals infected with *S*. *praecaptivus*. The presence of viable *S*. *praecaptivus* within progeny was verified via plating individual fly homogenates in selective media with identity confirmed through *tam* specific PCR. For each cross, the number of progeny positive for *S*. *praecaptivus/* total number of progenies sampled at that time point are provided. STZ, streptozotocin-treated. WT *S*. *praecaptivus* used was CD623 (S1 Table).

While WT *S*. *praecaptivus* infections persist long-term with no detectable impact on tsetse lifespan, we were interested in examining the effect of these infections on tsetse reproductive fitness. We first examined the impact of both parents harboring *S*. *praecaptivus* infections towards tsetse reproductive fitness since transmission of *S*. *glossinidius* can occur from either parent [39–42]. To accomplish this, both female and male tsetse were microinjected with 1 x 10^5^ WT *S*. *praecaptivus* and subsequently mated. The number of larvae deposited per mated female was determined weekly and compared to the fecundity of control flies (Fig 4A). Both *S*. *praecaptivus* infected and uninfected females began depositing pupae approximately one week following mating, indicating no lag in time to offspring deposition between the treatment groups. Further, comparison of the regression lines through the weekly fecundity of *S*. *praecaptivus* inoculated flies (Y = -0.001838*X + 0.1835) with that of controls (Y = 0.0009411*X + 0.4968), demonstrated no significant difference between slopes (Multiple linear regression, *p* = 0.9194), reflecting a constant reproductive rate in both groups. However, the number of larvae deposited per female was consistently higher in controls as supported by a significantly higher Y intercept for this group (Multiple linear regression, *p =* 0.0257). Despite a lower reproductive output, pupae deposited by the *S*. *praecaptivus-*infected parental line were similar in weight (0.02571 ± 0.0014g) to those deposited by the control group (0.02471 ± 0.0006g; Mann-Whitney test, *p* = 0.7466; Fig 4B). These results indicate that while tsetse flies inoculated with *S*. *praecaptivus* infection do reproduce successfully and produce viable and phenotypically normal offspring, their reproductive output is significantly lower, perhaps as a consequence of increased use of host (nutritional) resources by *S*. *praecaptivus*. Whether these *S*. *praecaptivus* infection costs are transgenerational merits further studies.

### *S*. *praecaptivus* is vertically transmitted in tsetse

**Fig 5:**
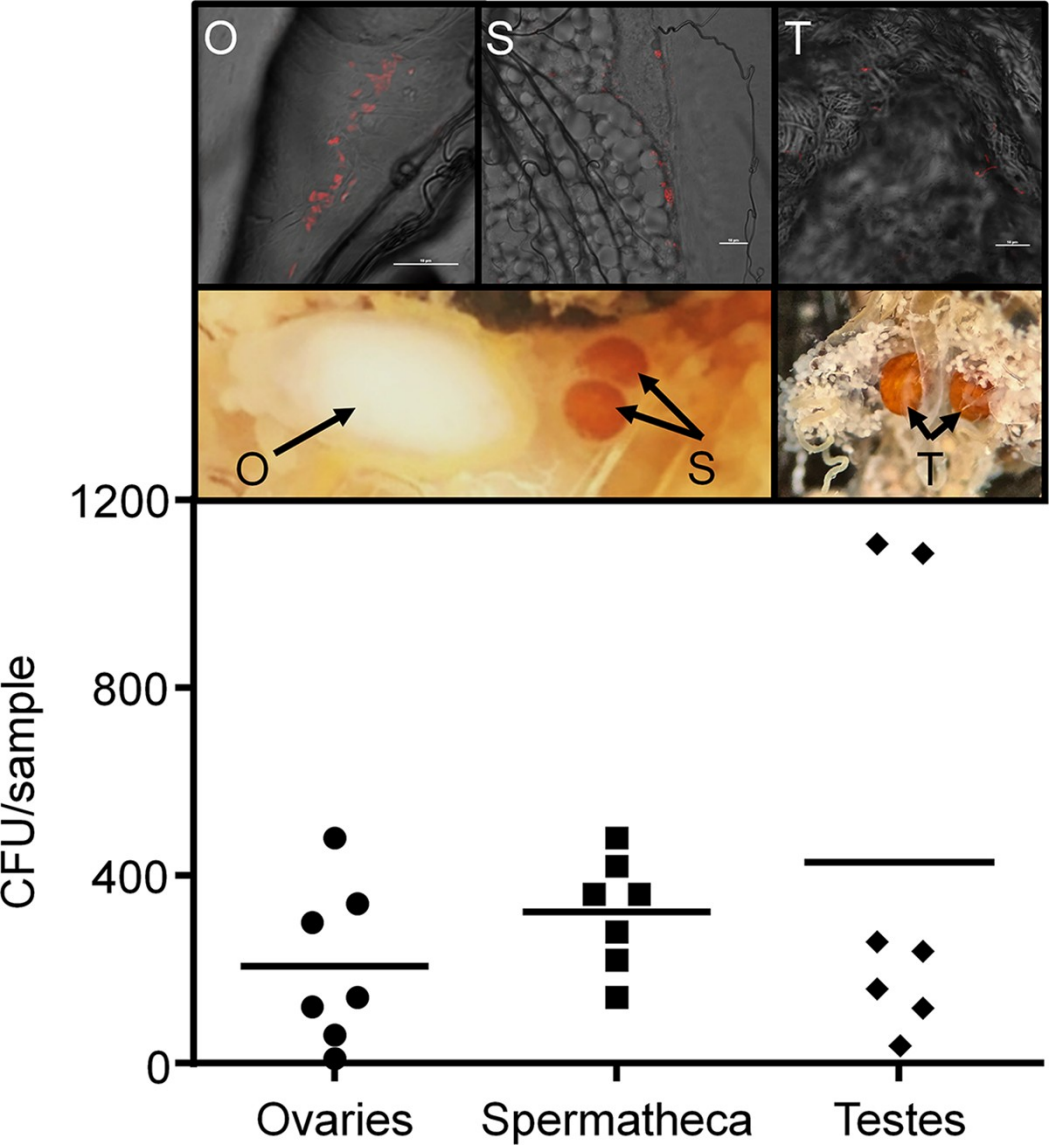
Colonization of tsetse reproductive tissue by *S*. *praecaptivus*. Tsetse individuals (unmated) received intrathoracic injections with 1 x 10^5^ WT *S*. *praecaptivus* (CD623, S1 Table) and 7 d post introduction reproductive tissues were dissected. Infections occurred in all sample types with no significant differences in density (ANOVA, *p* > 0.05). Horizontal lines represent mean CFU per treatment, while each dot represents the CFU within an individual. Insets above show mCherry-expressing *S*. *praecaptivus* invading the reproductive tissue of both sexes. O; ovary, S; spermathecae, T; testis. Scale bar is 10 μm.

We were interested in examining whether following establishment within tsetse, *S*. *praecaptivus* would undergo vertical transmission, a feature that is necessary for evolutionary persistence and/or development of mutualistic associations. To test the potential for vertical transmission within tsetse, WT *S*. *praecaptivus* was either injected or fed to females and/or males prior to mating. The resulting offspring were then tested for *S*. *praecaptivus* colonization at different points in pupal and adult development. The vertical transmission of *S*. *praecaptivus* was confirmed in three distinct tsetse lines at various times during development (Fig 4C); 1) males^STZ^ fed *S*. *praecaptivus* x females^WT^ (6.5% of offspring), 2) males^STZ^ injected with *S*. *praecaptivus* x females^STZ^ injected with *S*. *praecaptivus* (20% of offspring) and 3) males^WT^ injected with *S*. *praecaptivus* x females^WT^ injected with *S*. *praecaptivus* (19% of offspring). Notably, each of the successful combinations includes infected males and one of those includes uninfected females, indicating that paternal transmission took place. Although no transmission was observed under any conditions in which the male in the pairing was uninfected, it is notable that the highest frequency of infected progeny arose when both parents harbored *S*. *praecaptivus*. We found colonization of *S*. *praecaptivus* within testes, ovaries and spermathecae (i.e. female receptacle used for sperm deposit) within 100% of microinjected virgin flies. In additional support for the invasion of gonads, a mCherry-expressing *S*. *praecaptivus* localized to tsetse ovaries, spermathecae, and testes of microinjected flies. Comparable densities were found in these reproductive tissues suggesting that multiple routes of parental inheritance may enable *S*. *praecaptivus* transmission within tsetse (Fig 5). The vertical transmission of *S*. *praecaptivus* in tsetse is remarkable in the sense that the partners are naïve and have not been subject to an evolutionary opportunity to co-evolve. Moreover, this provides further evidence of the capacity of the *Sodalis*-allied symbionts to colonize a wide range of insects and develop mutualistic associations.

### Genes under quorum sensing control in both *S*. *glossinidius* and *S*. *praecaptivus*

**Fig 6:**
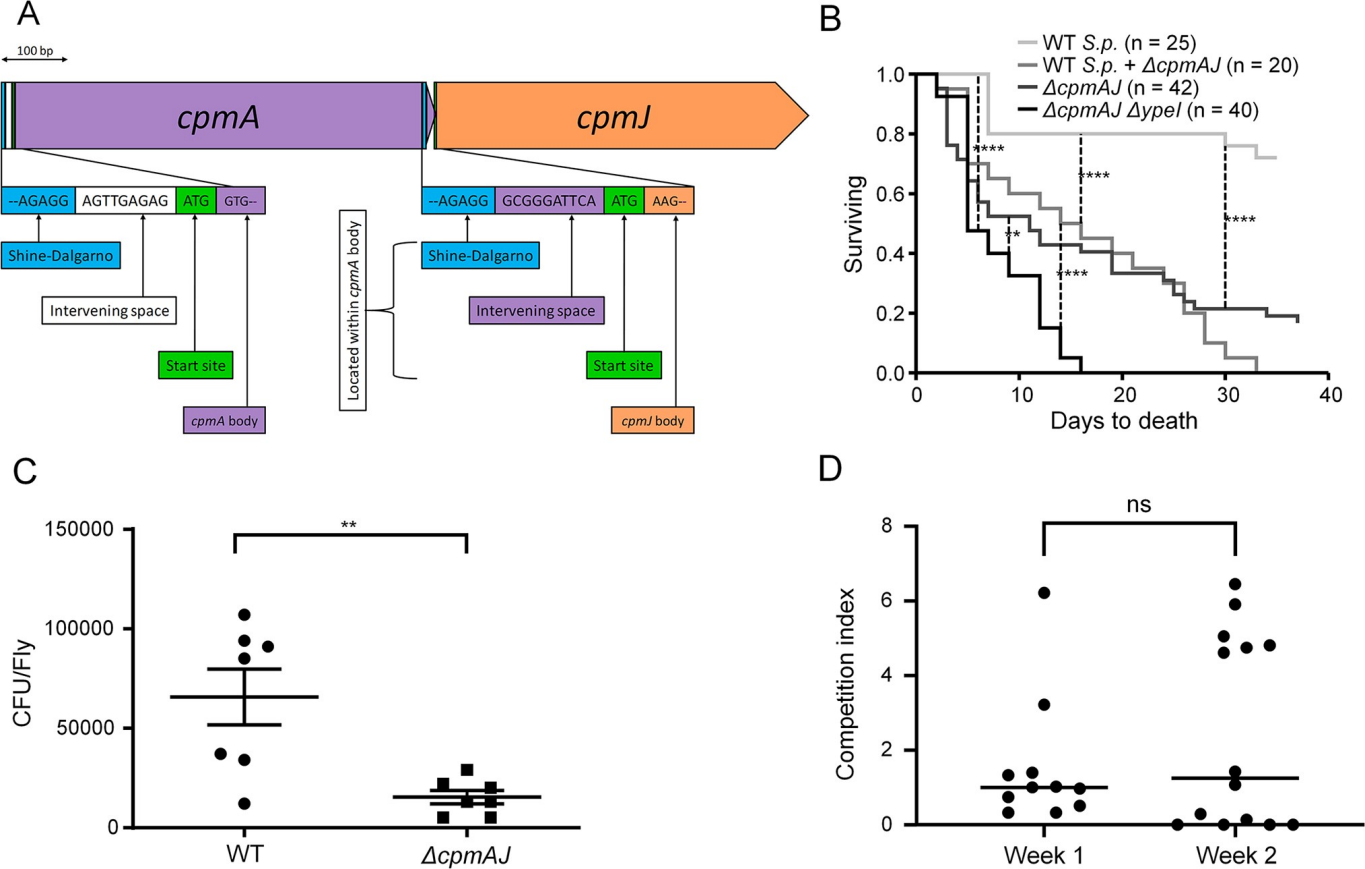
Homologs within carbapenem biosynthesis operon mediate attenuation of virulence in *S*. *praecaptivus*. A. Structure of *S*. *praecaptivus* genes *cpmA* and *cpmJ* (Sant_3163 and Sant_3164). B. In *S*. *praecaptivus*, disruption of QS regulated genes in the carbapenem biosynthesis pathway (*ΔcpmAJ*) increases virulence leading to lower tsetse survival: ** *p* ≤ 0.01, *** *p* ≤ 0.001, and **** *p* ≤ 0.0001. WT *S*. *praecaptivus* used for comparison was CD14 (S1 Table). C. The density of *S*. *praecaptivus* within flies following 20 d post introduction was assessed through CFU counts after surface sterilization, homogenization, serial dilution and plating of whole flies. Horizontal lines represent mean bacterial count, while dots represent mean bacterial count per individual. Mean bacterial density was compared (Student’s *t-*test, **, *p* = 0.0022). Bars represent 1 SEM. D. The *S*. *praecaptivus* mutant strain *ΔcpmAJ* does not outcompete the WT *S*. *praecaptivus* CD623. Competition index (CI) = (*ΔcpmAJ* output/WT output)/(*ΔcpmAJ* input/WT input). Horizontal lines represent mean CI between *S*. *praecaptivus ΔcpmAJ* and WT, while dots indicate the mean CI within individual flies.

Only truncated versions of *cpmA* (orthologs encode carbapenam-3-carboxylate synthase) and *cpmJ* (orthologs are involved in the resistance to carbapenem) are found in *Sodalis spp*. (S2 Table and Fig 6A). This gene retention pattern suggests a distinct functional role from antibiotic synthesis, which could be either ancestral to, or derived from, a complete carbapenem gene cluster that is capable of producing antibiotic [34]. Notably, *cpmA* and *cpmJ* are both under quorum sensing regulation, and unlike the virulence genes that are repressed under quorum, the expression of these genes is enhanced within both *S*. *glossinidius* and *S*. *praecaptivus* [27,34], which may indicate that it has an adaptive function in insect symbiosis.

In order to assess the importance of *cpmA* and *cpmJ* in the context of a nascent symbiosis, both genes were inactivated in *S*. *praecaptivus* and mutants were injected into tsetse. The survival of the *S*. *praecaptivus ΔcpmAJ* inoculated flies was significantly reduced in comparison with that of flies injected with the wildtype strain (Log-rank test, *p* < 0.0001; Fig 6B). Moreover, when a *ΔcpmAJ ΔypeI* triple mutant was introduced into tsetse, the survival of flies decreased even further (*ΔcpmAJ* vs. *ΔcpmAJ ΔypeI;* Log-rank test, p = 0.0024; Fig 6B). When *S*. *praecaptivus* abundance was determined in flies inoculated with either WT or *ΔcpmAJ* mutants (Fig 6C), a significantly higher bacterial density was found in flies harboring WT (Student’s *t*-test, *p* = 0.0044) indicating that host killing was occurring in the presence of only a small numbers of bacterial cells.

We were curious whether a WT strain of *S*. *praecaptivus* could reduce the virulence of the *ΔcpmAJ* strain upon mixed infection, in the same way that the WT strain can suppress the killing effect of the *ΔypeI* strain by providing a source of OHHL. However, injection of a 1:1 mixture of WT *S*. *praecaptivus* and the *ΔcpmAJ* mutant strain did not restore tsetse survival relative to flies injected with the *ΔcpmAJ* mutant alone ((WT + *ΔcpmAJ*) vs. *ΔcpmAJ*; Log-rank test, p = 0.5652; Fig 6B). However, tsetse inoculated with the mixture of WT and *ΔcpmAJ* mutant *S*. *praecaptivus* maintained equivalent levels of both of these strains at one and two week timepoints following injection (Fig 6D). Finally, we also tested the effect of the *ΔcpmAJ* mutant on grain weevil survival, to determine if it had a similar effect in those insects. Similar to what we observed in tsetse flies, a mutant in quorum sensing regulation (*ΔypeR*) was even more virulent when coupled with the *ΔcpmAJ* mutation (S4 Fig). This suggests that the *S*. *praecaptivus* CpmA and CpmJ proteins have a general protective effect in insects following infection. However, since little is known about the function of *cpmAJ* in *Sodalis* spp., further investigations are needed to determine how its activity limits virulence.

## Discussion

### Symbiosis follows diverse evolutionary paths

Upon entering a host, bacteria encounter specific physical and biological challenges that mandate the expression of specialized genes that facilitate bacterial survival. Establishment within a host requires sequestration of host resources for nutrition, resistance towards host immunity and (often) the secretion of virulence factors that facilitate entry of bacteria into host cells and tissues. While parasitic and mutualistic symbionts both need to establish access, overcome host defenses and obtain nutrition, mutualists have the added burden of limiting their virulence, in order to minimize negative impact on host fitness. In addition, mutualists need to evolve mechanisms to achieve vertical transmission, to ensure their maintenance in host offspring, providing natural selection with an opportunity to optimize traits that favor the persistence of the symbiotic partnership in nature [43].

### *S*. *praecaptivus* exhibits wide insect host adaptability

The current work, along with previous work, shows that *S*. *praecaptivus* is capable of establishing persistent infections in weevils and tsetse flies. The Coleoptera and Diptera orders belong to two major lineages of Holometabola, the Neuropteroidea and the Mecopterida, respectively. The divergence of these groups is estimated to have taken place in the Permian period, during the Paleozoic Era, around 300 million years ago [44]. Additionally, weevils and tsetse flies have widely different feeding habits, the former feeding on a variety of grains [45], while the latter consumes blood [46]. With such stark differences in host physiology and dietary ecology, it might be anticipated that a bacterium attempting to infect these hosts would face acutely distinct physiological environments that limit its ability to infect these diverse hosts. However, *S*. *praecaptivus* finds suitable nutrients for survival and acclimates to the microenvironmental physical constraints imposed by these divergent insect hosts. This highlights the potential of this bacterium to establish infection in a wide range of insect hosts, concordant with the observation that *Sodalis-*allied symbionts have been identified in a wide range of blood feeding and plant feeding insect taxa [25–27].

Our work shows that wild-type *S*. *praecaptivus* establishes a stable association in tsetse flies, as it does in grain weevils [27], characterized by a longstanding infection that has no detectable impact on insect longevity. However, our work also shows that *S*. *praecaptivus* mutants, lacking key components of the quorum sensing machinery, have a potent insect killing phenotype that is correlated with the loss of repression of virulence gene expression that is normally mediated by their quorum sensing gene regulatory system. Interestingly, host killing is mediated, at least in part, by PirAB toxins, which have been studied in other bacteria (notably *Photorhabdus spp*.) [33], but were not known to have such broad insecticidal activity.

### Quorum sensing attenuates virulence

The potential to infect a wide assortment of animal hosts is explained by the fact that the *S*. *praecaptivus* genome harbors many genes that are predicted to encode virulence factors and toxins believed (by virtue of homology) to target insect, plant and mammalian hosts. In terms of insect infections, specific factors including PirAB seem to serve to facilitate host establishment but are then suppressed transcriptionally, via quorum sensing based regulation, to enable bacterial persistence without negatively impacting host fitness [27]. Following establishment and vertical transmission, degenerative evolution is expected to catalyze inactivation and loss of genes that no longer have adaptive value in these bacterial-insect associations [25,47,48]. These adaptations evolve in accordance with functional mandates that are specific to a given host and are therefore anticipated to result in an ecological tradeoff that should also restrict the transfer of established symbionts into novel host species.

Virulence is a context-dependent trait that is influenced by bacterial infection dynamics [49] and the quantitative and qualitative mandate for toxin production [50]. On a basic level, parasites are anticipated to exhibit increased virulence relative to mutualists because their hosts are dispensable. On a more subtle level, mutualists are anticipated to minimize their virulence (and hosts are anticipated to exhibit immunotolerance towards their mutualistic microbial partners [51], so that energy is not wasted in unnecessary conflict. Thus, it makes sense that while genes encoding quorum sensing-regulated, insect-specific virulence factors (including *pirAB*) are maintained in a functional state in *S*. *praecaptivus* [27], their homologs are often found to be inactivated or lost in the genomes of the recently established mutualistic symbionts *S*. *glossinidius* [31] and *Ca*. S. pierantonius [47], both of which retain a functional quorum sensing system [34]. However, it is notable that both of those insect symbionts retain identifiable homologs of *cpmA* and *cpmJ*, which are among the few genes whose expression was demonstrated to be upregulated under quorum in *S*. *praecaptivus* [27] and *S*. *glossinidius* [34]. Strikingly, in this study, we show that the *cpmAJ* genes appear to have a beneficial (protective) function during *S*. *praecaptivus* infection of both weevils and tsetse flies but likely in distinct ways. Although the mechanism of this protective effect is not yet understood, it is interesting to note that tsetse flies inoculated with *ΔcpmAJ* mutants displayed increased mortality in the absence of significant bacterial proliferation. Based on the observation that the homolog of CpmJ found in carbapenem-synthesizing bacteria (CarG; an intrinsic carbapenem resistance protein) may function by modifying penicillin-binding proteins in the bacterial cell wall [52], it is conceivable that CpmAJ could modulate interactions between *S*. *praecaptivus* and the insect immune system, increasing host fitness [53]. In contrast, the *cpmAJ* mutant does not compromise survival, and bacterial density is comparable to wildtype *S*. *praecaptivus* densities within weevils. However, when we examined Δ*ypeR* Δ*cpmAJ*, we saw acceleration of host killing compared to Δ*ypeR* in weevils. This result indicates CpmAJ has a host protective effect in weevils as well. At this time, we do not know enough to speculate on the different roles of CpmAJ within distinct hosts.

### The ecological mandate for quorum sensing mediated virulence suppression

Our results demonstrate that the quorum sensing mediated control of *S*. *praecaptivus* virulence in tsetse flies operates in a remarkably similar way to that observed in grain weevils [27]. Based on the fact that these insects are so distantly related, it is tempting to speculate that quorum sensing mediated control of virulence would work in a wide range of insect hosts, explaining (at least in part) the widespread distribution of *Sodalis*-allied symbionts among insect taxa. However, because natural selection lacks foresight, it is important to recognize that the quorum sensing based modulation of virulence in *S*. *praecaptivus* must play an important (adaptive) role in the ecology of this bacterium in its free-living state. To this end, it has been suggested that *S*. *praecaptivus* is an opportunistic pathogen of plants and animals that may develop associations with insects in order to achieve vector-based transmission [25,27]. This is consistent with the observation that *S*. *praecaptivus* utilizes quorum sensing to limit its virulence towards insects based on the intuitive notion that natural selection will favor passengers that minimize the fitness costs associated with their transmission by vectors.

### Insight into the origins of *Sodalis*-insect associations

Because vectors will always be negatively impacted by the metabolic cost of maintaining a bacterial passenger, a trade-off might be anticipated to evolve in which the passenger provides the vector with some benefit that offsets the cost of maintenance, thereby sowing the seeds of a mutualistic relationship. Under circumstances in which there is a net benefit to the host, natural selection would then be expected to favor the evolution of a vertical transmission strategy [54]. In the case of many insect-bacterial mutualisms this is not difficult to rationalize on the basis that many free-living microbes are capable of synthesizing essential amino acids and vitamins that cannot be synthesized by insects and are lacking in certain diets such as blood or plant sap. As an alternative outcome to mutualism, other interactions may evolve such as parasitism driven by the enhanced fitness of the symbiont through host manipulation as observed with reproductive parasites.

Strikingly, our results show that, in addition to having a normal lifespan, tsetse flies infected with *S*. *praecaptivus* are capable of transmitting these bacteria vertically to their offspring (albeit at a relatively low frequency). Transmission events that were detected in our experiments always involved an infected male and could take place in the absence of an infected female. This indicates that transmission must have occurred via a strictly paternal route, as has been reported for *S*. *glossinidius* [42], but does not rule out the possibility that transmission can also occur via a maternal route. Moreover, both WT and mCherry-expressing *S*. *praecaptivus* cells were detected in tsetse ovaries, testes and spermathecae.

While more experimentation is needed to understand the dynamics of *S*. *praecaptivus* transmission in tsetse, our experiments do reveal that *S*. *praecaptivus* infection does incur a significant cost at the level of tsetse reproduction. Thus, in the absence of a significant compensatory benefit, flies infected with *S*. *praecaptivus* would be anticipated to be outcompeted by non-infected individuals. Obviously, our experiments were conducted in a laboratory setting in which there is no mandate for an adaptive benefit resulting from *S*. *praecaptivus* infection. However, in nature, associations are predicted to arise within the context of an opportunity for niche expansion. In addition, it stands to reason that the maintenance of a novel bacterial infection, with no adaptive benefit, would incur a fitness cost. Indeed, the acquisition and transmission of a novel bacterial partner is only expected to be beneficial if it facilitates adaptation towards environmental change, such as dietary niche expansion or resistance to a novel pathogen.

Clearly, the *Sodalis*-allied symbionts have been very successful in gaining entry into insects and evolving various symbiotic roles. However, we still understand little about how these associations originate in nature. Our results indicate that infection in tsetse flies can occur through oral introduction or hemocoel injection. Comparative genomic studies suggest that the establishment of *Sodalis*-allied symbionts has occurred independently in many insect hosts and our results certainly provide support for that notion. They also indicate that persistent infection can be maintained in the presence of existing *Sodalis*-allied symbionts, including *S*. *glossinidius* (in our study), where synergistic interactions between the two bacterial species may be occurring. For example, the production of OHHLs by *S*. *glossinidius* may enhance the number of *S*. *praecaptivus* in tsetse, although this remains speculative. This supports the notion that novel symbiont acquisitions could be added to augment the functions of existing symbionts, or that symbiosis can persist as a “revolving door”, in which existing symbionts that have been functionally compromised (by degenerative mutations) are refreshed by free-living candidates. Considering both of these scenarios, it seems important for those candidates to be able to persist and be transmitted efficiently. To that end, our work shows that *S*. *praecaptivus* has the ability to undergo vertical transmission in tsetse, albeit in a limited capacity and with negative impacts on host reproductive fitness. While we propose that this capability may help to explain the propensity of *Sodalis* spp. to develop associations with a wide range of insects, as observed in nature, it is important to recognize that natural selection has no ability (foresight) to act on future outcomes. Therefore, the ability of *S*. *praecaptivus* to undertake a cautious and controlled infection of insect hosts must have an adaptive benefit in the lifecycle of this bacterium prior to the evolution of stable associations.

### Application of *S*. *praecaptivus* to symbiosis research and paratransgenesis

Because of the ease of culture and genetic manipulation in the laboratory and its ability to infect a wide-range of insect hosts, *S*. *praecaptivus* represents an excellent candidate to study mechanistic aspects of symbiosis. The discovery that it is vertically transmitted in tsetse opens the door to discover and study the determinants of that mode of transmission through genetic and cell biological approaches. It also provides an opportunity to engineer *S*. *praecaptivus* strains that have increased efficiency of transmission and that phenocopy existing symbionts, such as those found in tsetse flies. This is particularly interesting in the context of paratransgenesis in which symbionts could be used to express genes that prevent insect vectors (such as tsetse, obligate vectors of African trypanosomes) from transmitting diseases of medical and agricultural importance. In the case of tsetse, such technology is envisioned to augment the sterile insect technique (SIT), in which irradiated male flies are released in the wild to mate with wild females, which then produce no offspring. One of the challenges with the implementation of SIT is that the release of the males can lead to an increase in disease transmission, because the sterile males are still competent to vector trypanosomes. Thus, it has been proposed that paratransgenic male tsetse flies, harboring symbionts that express anti-trypanosomal gene products, could be used to ameliorate this negative consequence [55]. Another challenge in this context is that the symbionts themselves need to be able to withstand the insect irradiation [56]. To this end, while *S*. *glossinidius* lacks a number of key DNA repair enzymes [27], *S*. *praecaptivus* naturally retains a full complement of this machinery, suggesting that it would be more robust in this application. Further, it should be noted that efficient vertical transmission is not a prerequisite for the implementation of this approach. Indeed, recombinant *S*. *praecaptivus* could be introduced to tsetse by pupal or adult microinjection prior to release.

### Conclusion

Our findings shed light on the molecular interplay taking place in the incipient stages of symbiosis, highlighting the versatility of quorum sensing towards facilitating adaptability and persistence of *S*. *praecaptivus* within dissimilar insect hosts. The novelty of our results is first rooted in providing support for *S*. *praecaptivus* being emblematic of a proto-symbiont for the *Sodalis* clade, particularly given its capability towards establishing in a wide host range. Secondly, we present evidence that quorum sensing is essential for transitioning from an environmental to a symbiotic state irrespective of the target host. Third, we demonstrate that the regulation of virulence through quorum sensing is fastened to an unprecedented ability to undergo vertical transmission quickly within a single host generation. Importantly, given the amenability towards genetic manipulation and its vertical transmission within tsetse, further optimization of *S*. *praecaptivus* towards paratransgenesis efforts could be used to bolster vector control strategies.

## Materials and methods

### Tsetse lines

The tsetse species, *Glossina morsitans*, is maintained in WVU’s insectary at 24°C with 50–55% relative humidity on a 12 h light:12 h dark photoperiod schedule. All flies receive defibrinated bovine blood (Hemostat, CA) every 48 h through an artificial membrane feeding system [57].

### *Sodalis praecaptivus* cultures and genetic manipulation

The *S*. *praecaptivus*^WT^ and mutant strains (S1 Table) were grown overnight with shaking at 200 rpm at 30ºC in LB media (lacking NaCl) with antibiotics as necessary [27]. When needed, antibiotics were added to media at the following concentrations: spectinomycin 40 μg/mL; gentamicin 5 μg/mL, kanamycin 25 μg/mL, tetracycline 5 μg/mL. Disruptions/deletions in *S*. *praecaptivus* genes were generated with the Lambda red recombineering system as reported previously (27).

### *Sodalis praecaptivus* mCherry mutant strain

The strain expressing mCherry was constructed using the Lambda red recombineering methods reported previously [27] using a construct comprising a Zeocin resistance cassette linked to an mCherry allele that was codon-optimized for expression in gamma-Proteobacteria [58,59].

### Introduction of *S*. *praecaptivus* into tsetse flies

The introduction of *S*. *praecaptivus* into tsetse flies was performed either through *per os* (i.e. oral introduction) into tenerals (i.e. newly emerged adults prior to acquiring a blood meal) or by microinjection of individuals following one blood meal to enable hardening of the cuticle prior to puncture. The introduction of *S*. *praecaptivus per os* involved feeding flies a heat-inactivated (56ºC for 30 min to destroy complement) blood meal which resulted in inoculations of 1.0 x 10^3^ or 1.0 x 10^5^ bacteria per fly. Flies that did not take a blood meal, determined through visual observation of tsetse abdomens, were excluded from analyses. The viability of the bacteria within heat-inactivated blood was confirmed by incubation for one week under the same conditions used to rear tsetse and then inoculating on L-plates (LB + 40 μg/mL X-Gal + 7 μg/mL IPTG, No NaCl) to confirm the presence of *S*. *praecaptivus* based on colony morphology and expression of ß-galactosidase. For microinjection, flies were first immobilized on ice and injected into the midthoracic region with needles immersed into overnight bacterial cultures. Microinjection resulted in approximately 1x10^5^ *S*. *praecaptivus* cells being introduced into each fly, as verified by plating needle contents. Following *S*. *praecaptivus* introduction, tsetse were returned to colony rearing conditions and fed defibrinated bovine blood with mortality recorded every 48 hours.

The establishment and density (CFU) of *S*. *praecaptivus* infections within tsetse was determined by surface sterilizing flies in 10% bleach for 5 min, homogenizing individuals in sterile deionized water, and plating a dilution series of the homogenates on L-plates. Colonies were confirmed to be *S*. *praecaptivus* through PCR amplification of the trans-aconitate 2-methyltransferase (*tam;* GenBank: AHF76984.1) gene using primers *tam*127 (5’-GCT ATT GGT CGA GCG TTT TAC C-3’) and *tam*128 (5’-CGG CAT CAC ATG GTA ATA GC-3’) with a 58°C annealing temperature.

### Tsetse reproductive fitness and *S*. *praecaptivus* infection

Following one blood meal, female and male tsetse received a thoracic microinjection of 1 X 10^5^ WT *S*. *praecaptivus*. Following 7 d post *S*. *praecaptivus* injection, challenged flies were mated with mortality and pupal deposition recorded every 48 hours and compared to age-matched mated controls. Tsetse reproductive fitness through time was determined by dividing the number of pupae deposited weekly by the number of mated females alive during each observation period.

### *S*. *praecaptivus* vertical transmission

Pupae obtained from mating crosses of *S*. *praecaptivus* challenged parents (as described above) were collected, weighed and reared individually. Pupae were randomly sampled through development to test for a viable *S*. *praecaptivus* infection by surface sterilization, homogenization and plating on selective media as described above. Colonies were verified as *S*. *praecaptivus* through PCR amplification of the *tam* gene.

Please refer to S1 Text for more information regarding generating *S*. *glossinidius*-free tsetse lines and the *S*. *praecaptivus* mCherry mutant strain. Information on fluorescence microscopy, competitive index and statistical analyses may be found in the S1 Text.

## Supporting information

S1 Text Additional Materials and Methods. (PDF)

S1 Fig Rescue effect on tsetse survival upon co-injection of ΔypeI mutant with an equivalent amount of *S. praecaptivus* WT. Kaplan-Meier curves comparing survival of tsetse lines injected with WT, *S*. *praecaptivus* ΔypeI, and a co-injection of ΔypeI and WT. There was no significant difference in tsetse survival between WT and those tsetse receiving the coinjection (p = 0.79). **** p < 0.0001. n = number of flies.
(PDF)

S2 Fig The density of *S. praecaptivus* within flies following 20 d post introduction was assessed through CFU counts after surface sterilization, homogenization, serial dilution and plating of whole flies. Horizontal lines represent mean bacterial count, while dots represent mean bacterial count per individual. Mean bacterial density was compared (ANOVA, Tukey’s multiple comparisons test **, p = 0.0022). Bars represent 1 SEM.
(PDF)

S3 Fig Symbiont infection status of progeny produced by Streptozotocin fed parental line. Lanes correspond to; 1, wildtype whole fly DNA; 2–8 whole fly DNA of streptozotocin line. Individuals used for 2–8 were from the fourth generation of the Streptozotocin-treated parental line, which was the generation used for examining the impact of endogenous *S*. *glossinidius* towards *S*. *praecaptivus* prevalence and density.
(PDF)

S4 Fig CpmAJ has a host protective function in weevils. Loss of YpeR and CpmAJ synergistically accelerate weevil demise. The difference between the two survival curves was statistically significant (Logrank test: *p =* 9.1 e-15).
(PDF)

S1 Table *S. praecaptivus* strains used in this study. (PDF)

S2 Table Percentage amino acid identity of *cpm* operon genes within Sodalis-allied insect symbionts. Bacteria identified as *Sodalis*-allied symbionts are considered [23]. The nucleotide database was searched manually for genes annotated as involved in the carbapenem biosynthesis pathway. As no genes were identified by this method, the canonical Cpm proteins from *Photorhabdus laumondii* subspp. *laumondii* TT01 were run via NCBI tBLASTn, against the nr nucleotide database. Protein IDs used include CpmA (CAE12477), CpmB (CAE12478), CpmC (CAE12479), CpmD (CAE12480), CpmE (CAE12481), CpmF (CAE12482), CpmG (CAE12483), CpmH (CAE12485) and CpmJ (CAE12486). Homology hits were included if these occurred within the *cpm* region. * Hits to CpmG and CpmJ were hitting in identical locations in the *S*. *glossinidius*, *S*. *praecaptivus* and *Ca*. S. pierantonius genomes. As additional verification, the truncated proteins from *Sodalis glossinidius* SG0585 (NCBI-PROTEIN ID BAE73860) and SG0586 (NCBI-PROTEIN ID BAE73860) were also used in comparisons. The e-value cutoff was set to 10–7.
(PDF)
