# Supplementary material for: Quorum sensing sets the stage for the establishment and vertical transmission of Sodalis praecaptivus in tsetse flies
Source: PLoS Genet. 2020 Aug 14;16(8):e1008992. doi: 10.1371/journal.pgen.1008992 (PMC7449468; doi:10.1371/journal.pgen.1008992)
Supplement: S1 Text — (PDF) [file pgen.1008992.s001.pdf]

MATERIALS AND METHODS

Tsetse flies lacking *S. glossinidius*. Tsetse flies that were cleared of endogenous *Sodalis glossinidius* were generated by collecting the pupae from a parental line maintained on streptozotocin (20 µg/mL) supplemented blood meals [1]. Random tsetse individuals (n=10) from the streptozotocin treated parental line are checked at each generation to verify the absence of *S. glossinidius* through PCR of whole fly DNA using oligonucleotides that amplify an internal transcribed spacer (ITS) region as described previously in [2]. *Sodalis*-free tsetse flies are maintained on separate feeding membranes from wild type flies.

***Sodalis praecaptivus* mCherry mutant strain.** The strain expressing mCherry was constructed using the Lambda red recombineering methods reported previously [3] using a construct comprising a Zeocin resistance cassette linked to an mCherry allele that was codon-optimized for expression in gamma-Proteobacteria [4, 5]. This construct was modified for recombination by adding flanking sequences that target the lacZ gene in *S. praecaptivus*, based on the notion that lactose utilization is dispensable for insect association. The mCherry-zeocin cassette was first amplified by PCR in a reaction composed of 10µl 5X PCR buffer (Thermo Fisher Scientific), 4µl 25mM dNTPs, 3µl 25mM $MgCl_2$, 1.25µl of 20µM forward primer (5'-CTTCTTAAACATAAAGTGTCTC-3'), 1.25µl of 20µM reverse primer (5'-GACAGTCATTCATCTTTCTGC-3'), 0.5µl of Phusion High Fidelity DNA Polymerase (Thermo Fisher Scientific) and 1µl of purified template DNA. The thermocycling was performed using a 2 min denaturation at 98°C followed by 29 cycles of 98°C for 30 s, 56°C for 30 s, 72°C for 1 min and a final extension at 72°C for 2 mins.

To generate the recombination construct, 212 bp of the 5' end (5'-AAGTCACACGCTCACACCAG-3'/5'-GTTTATAAGGAGACACTTTATGTTTAAGAAGACGTGGTTGCACGTAAATGA-3') and 278bp of the 3' end (5'-CTTTTGGAGGGGCAGAAAGATGAATGACTGTCTTGACCGAGACAGCTCATTG-3'/5'-TCAGCATCGCAGTCTTCATC-3') of the *S. praecaptivus lacZ* gene were amplified with flanking tails using the following PCR reactants: 12.5µl of 2X Phusion PCR buffer, 6.5µl of nuclease free water, 2.5µl of 2.5µM forward primer, 2.5µl of 2.5µM reverse primer and 1µl of *S. praecaptivus* WT DNA. The PCR

was performed using an initial denaturation of 98°C 30 s followed by 35 cycles of 98°C for 10 s, 58°C for 30 s and 72°C for 2 min. The resulting PCR products were then purified using Agencourt AMPure XP beads, according to the product instructions. Four microliters of the purified products were then combined with 4ul of the mCherry-Zeo^R amplicon and 12µl of 2X Taq Polymerase MasterMix (Thermo Fisher Scientific) and subject to initial denaturation at 95°C for 30 s followed by 10 cycles of 94°C for 15 s, 45 °C for 30 s and 72 °C for 1 min. The final desired recombination construct was amplified with 1µl each of forward (5'-AAGTCACACGCTCACACCAG-3') and reverse primers (5'-TCAGCATCGCAGTCTTCATC-3'), 25 µl of 2X Taq Polymerase MasterMix (Thermo Fisher Scientific), and 23 µl of the PCR product from the 10-cycle reaction using an initial denaturation at 95°C for 30 s, followed by 35 cycles of 94°C for 15 s, 58°C for 30 s and 72°C for 1.5 min. The PCR product was then purified using Agencourt AMPure XP beads.

For Lambda-Red recombineering, 2.5 ml of an overnight *S. praecaptivus* strain 101 culture which maintains a plasmid with inducible Lambda-Red recombination functions was grown in 25 ml 2YT 5.8 media (20 mg/mL Tryptone, 8 mg/mL Yeast Extract, 10 mg/mL NaCl, pH 5.8) with 25 µg/ml chlorophenicol for 8 h at 30 ºC. The expression of the Lambda-Red functions was then induced by adding 500 µl of 20% arabinose to the culture and allowing it to grow for another 30 min. The bacteria were then centrifuged (8,000 X g, 5 min) and washed in cold sterilized DI water twice to make them electrocompetent. The 50 µl of prepared disruption fragment was then transferred into the electrocompetent cells by electroporation at 1600V/s using an Eppendorf electroporator model 2510. The cells were permitted to recover for 8-10 h recovery on an L plate at 30 ºC and then replica plated onto an L plate with 15 µg/ml zeocin and IPTG/X-gal to select for lac disruption (i.e. lac$^-$, zeocin^R). Recombinant colonies demonstrated a pink appearance as a consequence of *lacZ* inactivation and mCherry expression. They were then validated by PCR and sequencing and subjected to fluorescence microscopy to ensure mCherry expression.

Fluorescence microscopy. Gonads and spermathecae from virgin flies 7 d following microinjection of *S. praecaptivus* mCherry mutant strain were dissected in Phosphate Buffered Saline and placed on

microscope slides. Images were obtained using an inverted Nikon A1R confocal with a 60X/1.4 plan Apo oil objective. The *S. praecaptivus* mCherry mutant strain was detected in the PE-Texas Red Channel with a 610/20 bandpass filter.

Competitive index. Flies were microinjected with a 1:1 volume mixture of overnight cultures of wildtype (WT) *S. praecaptivus* and an *S. praecaptivus* null mutant lacking the genes *cpmA* and *cpmJ* that are known to be upregulated by QS [3]. Bacterial densities were assessed one and two weeks post introduction. Flies were surface sterilized, homogenized, and serially diluted. Dilution series were plated on non-selective media, while an equivalent amount was also plated on selective media (L-plates supplemented with gentamicin) and counted following overnight growth. Both strains are capable of growth on non-selective media, while only the *ΔcpmAJ* strain is able to grow on gentamicin. The colony count of WT *S. praecaptivus* was obtained by subtracting the colony count in the selective plate from the non-selective plate for each fly analyzed. The competitive index was calculated as follows:

$$Competitive\ index = \frac{mutant\ output/competitor\ output}{mutant\ input/competitor\ input}$$

Where mutant is the *ΔcpmAJ* strain, competitor is the *S. praecaptivus* wildtype strain, *input* is the bacteria count injected and *output* is the bacteria count recovered on plates [6].

Statistical analyses. As appropriate, statistically significant differences between treatment groups were identified with a *t*-test, non-parametric Mann-Whitney test or one-way ANOVA with Bonferroni correction for multiple comparisons using Prism version 8.1.2 (GraphPad Software, CA). Tsetse survival curves were created using the Kaplan-Meier method and statistically analyzed using the log-rank test [7] with JMP software version 10.0.1 (SAS Institute, USA). Least squares regression lines for comparing the fecundity of control and WT *S. praecaptivus* injected flies were obtained through multiple regression analysis using Prism.

References cited

1. Dale, C. and S.C. Welburn, *The endosymbionts of tsetse flies- manipulating host-parasite interactions.* International Journal of Parasitology, 2001. **31**: p. 628-631.
2. Snyder, A.K., Adkins, K.Z. and R.V.M. Rio, *Use of the Internal Transcribed Spacer (ITS) regions to examine symbiont divergence and as a diagnostic tool for Sodalis-related bacteria.* Insects, 2011. **2**: p. 515-531.
3. Enomoto, S., et al., *Quorum Sensing Attenuates Virulence in Sodalis praecaptivus.* Cell Host Microbe, 2017. **21**(5): p. 629-636 e5.
4. Balleza, E., J.M. Kim, and P. Cluzel, *Systematic characterization of maturation time of fluorescent proteins in living cells.* Nat Methods, 2018. **15**(1): p. 47-51.
5. Kim, J.M., et al., *Stochastic transcriptional pulses orchestrate flagellar biosynthesis in.* Sci Adv, 2020. **6**(6): p. eaax0947.
6. Silver, A.C., et al., *Interaction between innate immune cells and a bacterial type III secretion system in mutualistic and pathogenic associations.* Proc Natl Acad Sci U S A, 2007. **104**(22): p. 9481-6.
7. Bewick, V., L. Cheek, and J. Ball, *Statistics review 12: survival analysis.* Crit Care, 2004. **8**(5): p. 389-94.
