# Supplementary figures and images for: Quorum sensing sets the stage for the establishment and vertical transmission of Sodalis praecaptivus in tsetse flies
Source: PLoS Genet. 2020 Aug 14;16(8):e1008992. doi: 10.1371/journal.pgen.1008992 (PMC7449468; doi:10.1371/journal.pgen.1008992)

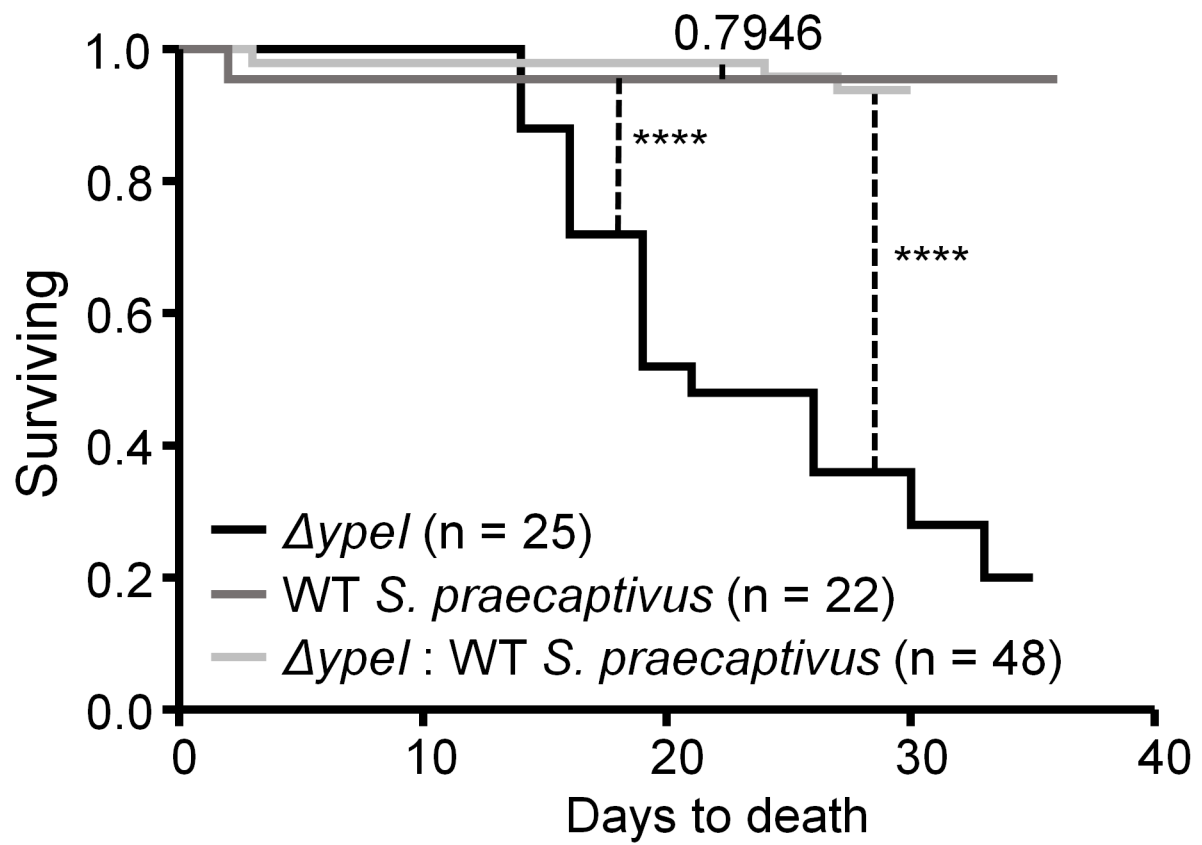

S1 Fig

Supplement: S1 Fig — Kaplan-Meier curves comparing survival of tsetse lines injected with WT, S. praecaptivus ΔypeI, and a co-injection of ΔypeI and WT. There was no significant difference in tsetse survival between WT and those tsetse receiving the coinjection (p = 0.79). **** p < 0.0001. n = number of flies. (PDF) [file pgen.1008992.s002.pdf]

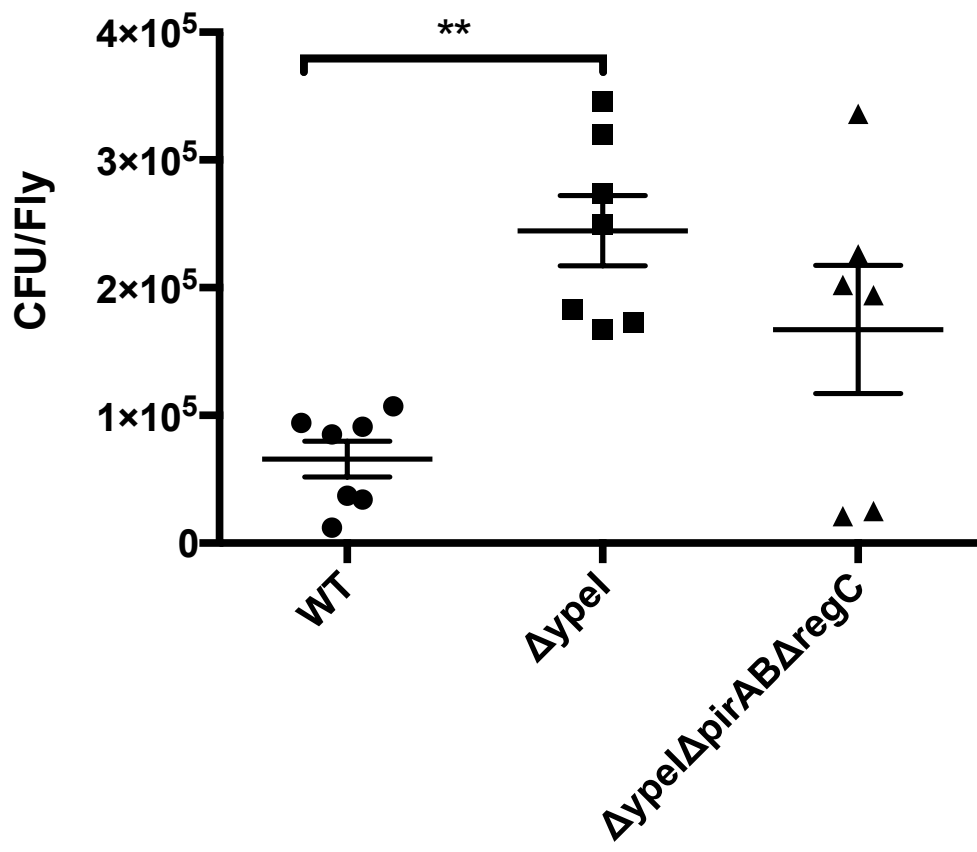

S2 Fig

Supplement: S2 Fig — Horizontal lines represent mean bacterial count, while dots represent mean bacterial count per individual. Mean bacterial density was compared (ANOVA, Tukey’s multiple comparisons test **, p = 0.0022). Bars represent 1 SEM. (PDF) [file pgen.1008992.s003.pdf]

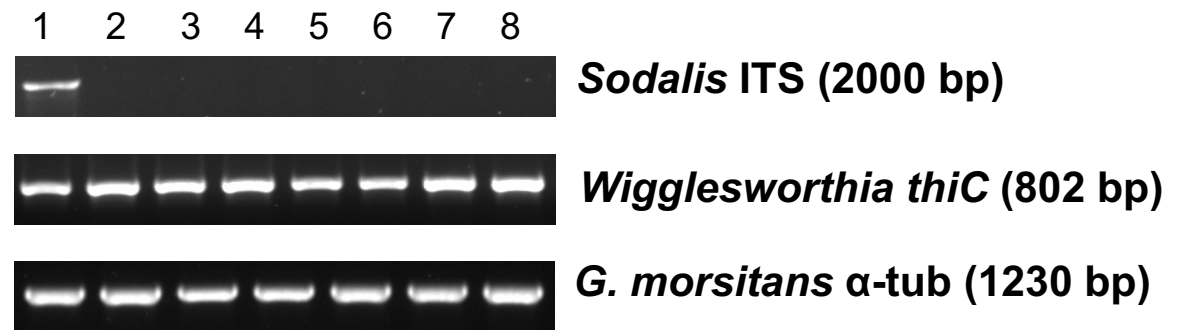

S3 Fig

Supplement: S3 Fig — Lanes correspond to; 1, wildtype whole fly DNA; 2–8 whole fly DNA of streptozotocin line. Individuals used for 2–8 were from the fourth generation of the Streptozotocin-treated parental line, which was the generation used for examining the impact of endogenous S. glossinidius towards S. praecaptivus prevalence and density. (PDF) [file pgen.1008992.s004.pdf]

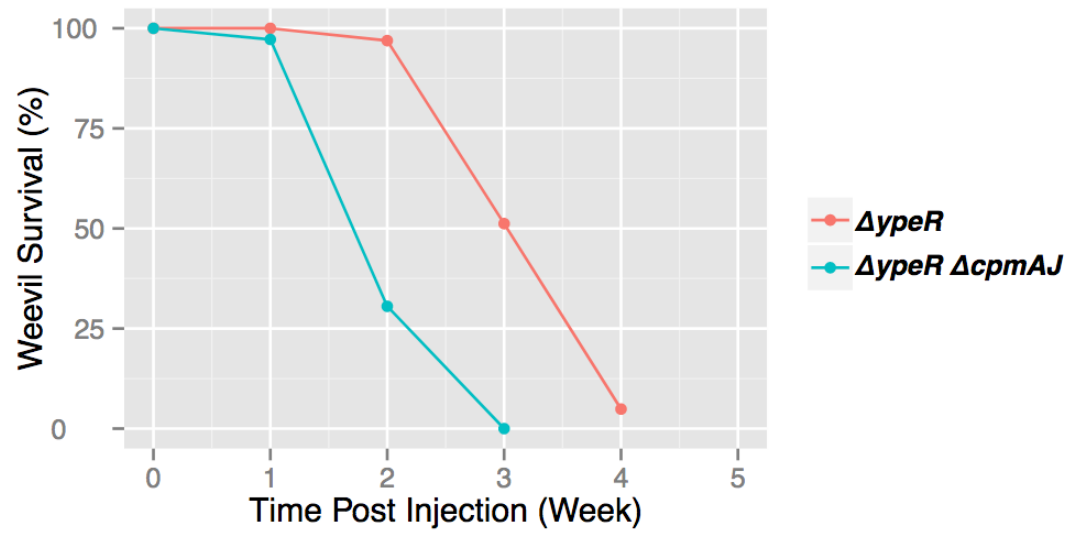

S4 Fig

Supplement: S4 Fig — Loss of YpeR and CpmAJ synergistically accelerate weevil demise. The difference between the two survival curves was statistically significant (Logrank test: p = 9.1 e-15). (PDF) [file pgen.1008992.s005.pdf]
