# Supplementary material for: Quorum sensing sets the stage for the establishment and vertical transmission of Sodalis praecaptivus in tsetse flies
Source: PLoS Genet. 2020 Aug 14;16(8):e1008992. doi: 10.1371/journal.pgen.1008992 (PMC7449468; doi:10.1371/journal.pgen.1008992)
Supplement: S1 Table — (PDF) [file pgen.1008992.s006.pdf]

S1 Table: *S. praecaptivus* strains used in this study.

**Strain**	**Features**	**Mutated gene (NCBI-Protein ID): protein encoded [reference]**
CD345	*S. praecaptivus ΔcpmAJ*::Gen	*cpmAJ* (AHF78165 and AHF78166): Putative carbapenem biosynthesis and resistance proteins, respectively [41].
CD348	*S. praecaptivus ΔcpmAJ*::Gen *ΔypeI*::Spc	*cpmAJ* (AHF78165 and AHF7816664): Putative carbapenem biosynthesis and resistance proteins, respectively / *ypeI* (AHF78570): Acyl-homoserine-lactone synthase [41].
CD286	*S. praecaptivus ΔypeR*::Spc	*ypeR* (AHF78569): Transcriptional activator of quorum-sensing system [41].
CD623	*S. praecaptivus Δsant_2908*::Tet	*hicA* (AHF77918): mRNA interferase; toxin-antitoxin system, strain used as WT with tetracycline resistance [this study].
CD1816	*S. praecaptivus ΔcpmAJ*::Gen *ΔypeR*::Spc	*cpmAJ* (AHF78165 and AHF78166): Putative carbapenem biosynthesis and resistance proteins, respectively /*ypeR* (AHF78569): Transcriptional activator of quorum-sensing system [this study].
CD433	*S. praecaptivus ΔypeR*::Gen *ΔyenR*::Spc	*ypeR* (AHF78569): Transcriptional activator of quorum-sensing system [this study]/*yenR* (AHF76244): Transcriptional regulator LuxR family [41].
CD384	*S. praecaptivus ΔyenR*::Spc	*yenR* (AHF76244): Transcriptional regulator of the LuxR family [41].
CD857	*S. praecaptivus ΔypeI*::Spc *ΔpirAB*::Gen *ΔregC*::Kan	*ypeI* (AHF78570): Acyl-homoserine-lactone synthase [41]/ *pirAB* (AHF77486 and AHF77486): insecticidal binary toxin complex PirAB / *regC* (AHF78621) homolog of a bacteriophage P2 transcriptional repressor RegC
CD298	*S. praecaptivus ΔypeI*::Spc	*ypeI* (AHF78570): Acyl-homoserine-lactone synthase [41]
CD14	*S. praecaptivus* wild-type	No mutation was introduced
