# Supplementary material for: Quorum sensing sets the stage for the establishment and vertical transmission of Sodalis praecaptivus in tsetse flies
Source: PLoS Genet. 2020 Aug 14;16(8):e1008992. doi: 10.1371/journal.pgen.1008992 (PMC7449468; doi:10.1371/journal.pgen.1008992)
Supplement: S2 Table — Bacteria identified as Sodalis-allied symbionts are considered [23]. The nucleotide database was searched manually for genes annotated as involved in the carbapenem biosynthesis pathway. As no genes were identified by this method, the canonical Cpm proteins from Photorhabdus laumondii subspp. laumondii TT01 were run via NCBI tBLASTn, against the nr nucleotide database. Protein IDs used include CpmA (CAE12477), CpmB (CAE12478), CpmC (CAE12479), CpmD (CAE12480), CpmE (CAE12481), CpmF (CAE12482), CpmG (CAE12483), CpmH (CAE12485) and CpmJ (CAE12486). Homology hits were included if these occurred within the cpm region. * Hits to CpmG and CpmJ were hitting in identical locations in the S. glossinidius, S. praecaptivus and Ca. S. pierantonius genomes. As additional verification, the truncated proteins from Sodalis glossinidius SG0585 (NCBI-PROTEIN ID BAE73860) and SG0586 (NCBI-PROTEIN ID BAE73860) were also used in comparisons. The e-value cutoff was set to 10–7. (PDF) [file pgen.1008992.s007.pdf]

S2 Table. Percentage amino acid identity of *cpm* operon genes within *Sodalis*-allied insect symbionts.

Bacteria identified as *Sodalis*-allied symbionts are considered [1]. The nucleotide database was searched manually for genes annotated as involved in the carbapenem biosynthesis pathway. As no genes were identified by this method, the canonical *cpm* proteins from *Photorhabdus laumondii* subspp. laumondii TT01 were run via NCBI tBLASTn, against the nr nucleotide database. Protein IDs used include CpmA (CAE12477), CpmB (CAE12478), CpmC (CAE12479), CpmD (CAE12480), CpmE (CAE12481), CpmF (CAE12482), CpmG (CAE12483), CpmH (CAE12485) and CpmJ (CAE12486). Homology hits were included if these occurred within cpm region. * Hits to CpmG and CpmJ were hitting in identical locations in the *S. glossinidius*, *S. praecaptivus* and Ca. *S. pierantonius* genomes. As additional verification, the truncated proteins from *Sodalis glossinidius* SG0585 (NCBI-PROTEIN ID BAE73860) and SG0586 (NCBI-PROTEIN ID BAE73860) were also used in comparisons. The e-value cutoff was set to 10^{-7}.

**Species**	**Taxonomy Id**	**CpmA**	**CpmB**	**CpmC**	**CpmD**	**CpmE**	**CpmF**	**CpmG***	**CpmH**	**CpmJ***	**SG0586**	**SG0585**
Mikella endobia	1778264	-	-	-	-	-	-	-	-	-	-	-
Moranella endobia PCVAL	1234603	-	-	-	-	-	-	-	-	-	-	-
Ca. *Moranella endobia* PCIT	903503	-	-	-	-	-	-	-	-	-	-	-
Ca. *Hoaglandella endobia*	1778263	-	-	-	-	-	-	-	-	-	-	-
Ca. *Doolittlea endobia*	1778262	-	-	-	-	-	-	-	-	-	-	-
Ca. *Gullanella endobia*	1070130	-	-	-	-	-	-	-	-	-	-	-
S-endosymbiont of *Heteropsylla cubana*	134287	-	-	-	-	-	-	-	-	-	-	-
S-endosymbiont of *Ctenarytaina eucalypti*	1199245	-	-	-	-	-	-	-	-	-	-	-
Sodalis-like symbiont of *Philaneus spumarius* PSPU	1273402	-	-	-	-	-	-	-	-	-	-	-
Candidatus *Zinderia insecticola* CARI	884215	-	-	-	-	-	-	-	-	-	-	-
P-endosymbiont of *Henestaris halophilus*	1929246	-	-	-	-	-	-	-	-	-	-	-
Sodalis-like endosymbiont of *Proechinophthirus fluctus* str. SPI-1	1462730	-	-	-	-	-	-	-	-	-	-	-
Sodalis glossinidius str. “*morsitans*”	1173031	25.48	-	-	-	-	-	27.78	-	27.78	100	99
Ca. *Sodalis melophagi*	343509	-	-	-	-	-	-	-	-	-	-	-
Sodalis praecaptivus	1239307	25.49	-	-	-	-	-	26.67	-	26.67	93.33	86
Ca. *Sodalis pierantonius* str. SOPE	2342	25.49	-	-	-	-	-	32.08	-	32.08	92.38	88

SI References

1. Santos-Garcia, D. et al., *The All-Rounder Sodalis: A New Bacteriome Associated Endosymbiont of the Lygaeoid Buig Henesteris halophilus (Heteroptera: Henestarinae) and a critical examination of its evolution.* Genome Biol Evol, 2017. **9**(10): 2893-2910.
